# Cyclin alterations in diverse cancers: outcome and co-amplification network

**DOI:** 10.18632/oncotarget.2848

**Published:** 2014-12-18

**Authors:** Maria Schwaederlé, Gregory A. Daniels, David E. Piccioni, Paul T. Fanta, Richard B. Schwab, Kelly A. Shimabukuro, Barbara A. Parker, Razelle Kurzrock

**Affiliations:** ^1^ Center for Personalized Cancer Therapy, and Division of Hematology and Oncology, UCSD Moores Cancer Center, La Jolla, USA

**Keywords:** cyclin, next generation sequencing, molecular profile, amplification, Cancer

## Abstract

Cyclin genes are key regulatory components of the cell cycle. With the development of new agents, cyclin-related genes are becoming increasingly important as they can be targeted. Yet, the biological implications of these alterations have not been fully studied. Clinical characteristics and outcome parameters were compared for patients harboring cyclin alterations versus not. *CCN* alterations were found in 13% of our population (50/392; all amplifications) and were associated with breast cancer (*P* < 0.0001), a higher median number of concomitant molecular alterations (*P* < 0.0001), and liver metastases (*P* = 0.046). Harboring a cyclin amplification was not associated with overall survival, the time to metastasis/recurrence, nor with the best progression-free survival. In a Cox regression model, gastrointestinal histology (*P* < 0.0001)*, PTEN (*P* < 0.0001)*, and *CDK* alterations (*P* = 0.041) had a significant association with poorer overall survival. *CCN* amplifications significantly correlated with alterations in *FGF/FGFR* family genes as well as in *MET* and *ARFRP1*. An extended correlation study shed light on a network of co-amplifications influenced in part by genes that were localized on the same amplicons. *CCN* amplifications are common across cancers and had distinctive biological associations. Customized combinations targeting the cyclin pathway as well as the extended co-amplification network may be necessary in order to address resistance mechanisms.

## INTRODUCTION

**Figure 1:**
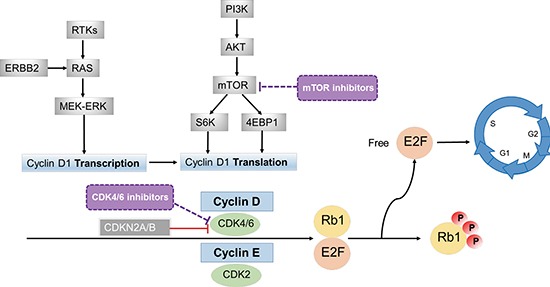
Effects of cyclins on cell cycle By interacting with CDKs, cyclins form complexes (cyclin D with CDK4/6 and cyclin E with CDK2) that phosphorylate Rb1 (phosphorylated Rb1 is inactive). The Rb protein is a tumor suppressor that plays a pivotal role in the negative control of the cell cycle; Rb1 loss of function is frequently observed in cancer [48]. When Rb1 is phosphorylated, E2F is released and can transcriptionally activate its target genes, enabling the G1/S transition of cell cycle. Cyclin D1 (CCND1) can also be regulated at the transcription level by the RAS-MEK-ERK pathway and at the translation level by mTOR via S6K and 4EBP1 [11, 12, 16, 17]. mTOR inhibitors may attenuate CCND1 action by decreasing translation of CCND1. CDK4/6 inhibitors may attenuate the effects of this pathway as well, especially in the presence of CDK4/6 amplification or CDKN2A/B loss (since CDKN2A/B inhibits CDK4/6 activity). Patients with Rb1 loss or mutations would be expected to be resistant to agents such as mTOR or CDK4/6 inhibitors that act more proximally.

Cyclins are key regulatory components of the cyclin/CDK (Cyclin-Dependent Kinase) complex that regulate the cell cycle, thus contributing to tumor progression. While cyclin D interacts with CDK4/6, cyclin E interacts with CDK2 to form complexes that play a central role in the G1/S transition of the cell cycle. This complex formed by cyclins and CDKs act by phosphorylating Rb [1, 2], which releases E2F from the complex, allowing it to activate cell cycle progression, Figure 1. Amplifications of cyclin genes are amongst the most common alterations in cancers, with *cyclin D1* (*CCND1*) amplification rates ranging from 15–40% [1].

*Cyclin D1* or *CCND1* was initially designated *PRAD1* or *BCL1*. *PRAD1* was found to be clonally rearranged on chromosome 11q13 with *PTH* (parathyroid hormone) in parathyroid adenomas, resulting in overexpression of cyclin D1 [3–5]. Similarly, in mantle cell lymphomas, *BCL-1* (B-Cell lymphoma-1) is rearranged and juxtaposes *BCL-1* and the immunoglobulin heavy chain *IGH* (t(11;14)(q13;q32)) resulting in overexpression of the BCL-1 (CCND1) protein [6, 7].

*CCND1* amplifications have been described in head and neck carcinomas, non-small-cell lung, endometrial, pancreatic, breast, as well as colorectal cancers [1]. Of interest, in addition to direct *CCND1* amplification, and rearrangement as described above, CCND1 can be overexpressed through several other mechanisms. Activation of the RAS-MEK-ERK pathway, along with ERBB2 in hormonal-driven cancers (such as breast) and the Wnt pathway [8–10] have also been associated with higher CCND1 expression, that may occur through increasing its transcription [11, 12]. The PI3K-AKT-mTOR pathway also regulates *CCND1* translation mechanisms [13–15] via mTOR and phosphorylation of downstream targets such as 4EBP1 (eukaryotic translation initiation factor 4E) and S6K (ribosomal protein S6 kinase) [16, 17] (Figure 1).

Cyclins have become increasingly relevant in cancer because drugs that can mitigate their effects are now available. Possible approaches to targeting cyclin-dependent cancers include use of CDK4/CDK6 inhibitors, either alone or in combination. Another approach might include use of mTOR inhibitors, since suppressing mTOR would attenuate the translation of *CCND*1 [18, 19]. Of interest in this regard, the mTOR inhibitor temsirolimus is approved in the European Union for the treatment of mantle cell lymphoma, a disease whose hallmark is *CCND1/BCL-1* translocation, resulting in overexpression of CCND1 [20].

Though cyclin gene (*CCN*) amplifications are frequently observed in different cancers [1], the biological and clinical implications of these alterations have not been comprehensively studied. Hence, we used next generation sequencing in a population comprising 392 patients with cancer, aiming to elucidate the correlation between *CCN* alterations with clinical characteristics and outcome.

## RESULTS

### Patient characteristics

The clinical and demographic characteristics of our patient population (*N* = 392) can be found in Table 1. The median age of patients at diagnosis was 54 years (CI 95%, 53–56). Women comprised 57% (*N* = 222) of the population. The majority of patients were Caucasian (72%, *N* = 284). The most represented tumor types were gastro-intestinal (23%, *N* = 91), breast (21%, *N* = 81), and brain (14%, *N* = 56) cancers. The molecular testing was performed mainly on the primary tumor (59% of patients, *N* = 218).

**Table 1:** Clinical characteristics of 392 patients with or without *CCN* amplifications (univariate analysis)

Characteristics	Total patients, *N* = 392	CCN wild-type, *N* = 342	CCN amplified, *N* = 50	*P*-value
**Age at diagnosis (years)**	54.3	54.4	52.5	0.860
(Median, CI 95%)	(52.5–56.0)	(52.6–56.3)	(48.8–58.1)	
**Gender**				**0.009**
Women	222	185 (83%)	37 (17%)	
Men	170	157 (92%)	13 (8%)	
**Race**				
Caucasian	284	250 (88%)	34 (12%)	0.498
Other	57	47 (82%)	10 (18%)	
Asian	25	22 (88%)	3 (12%)	0.281
African American	12	12 (100%)	0	
Unknown	10	8 (80%)	2 (20%)	
Hispanic	4	3 (75%)	1 (25%)	
**Type of cancer**				
Gastro-intestinal	91	81 (89%)	10 (11%)	0.720
Breast	81	59 (73%)	22 (27%)	**< 0.0001**
Brain	56	56 (100%)	0	**0.0004**
Gynecologic	33	31 (94%)	2 (6%)	0.287
Head and neck	30	25 (83%)	5 (17%)	0.566
Liquid	30	28 (93%)	2 (7%)	0.402
Melanoma	29	27 (93%)	2 (7%)	0.560
Lung	26	21 (81%)	5 (19%)	0.355
Other^a^	16	14 (88%)	2 (12%)	1.000
**Biopsy site used for molecular testing^b^:**				**0.029**
Primary	218	196 (90%)	22 (10%)	
Metastatic	149	122 (82%)	27 (18%)	

a Ewing sarcoma, carcinoid tumor, sarcomatoid tumor, peripheral nerve sheath tumor, pleiomorphic cell sarcoma (thigh), soft tissue liposarcoma (*N* = 2), soft tissue rhabdomyosarcoma, pleomorphic liposarcoma, and unknown origin (*n* = 7).

b For 25 samples, the origin of the biopsy site used for molecular testing was unavailable.

### *CCN* alterations and associations with clinical features

Overall, altered cyclin genes (*CCND1, CCND2, CCND3*, and *CCNE1*) were observed in 50 of the 392 patients tested (13%). There were a total of 53 *CCN* alterations found in 50 patients (three patients had two alterations), Supplemental Figure 1. All the *CCN* alterations identified were amplifications; the most frequent were *CCND1* amplifications (55%, *N* = 29/53). In a univariate analysis, *CCN* alterations were significantly associated with women (17% vs 8%, women:men; *P* = 0.009). *CCN* alterations were also associated with breast cancer (*N* = 22/81 (27%) of breast cancer cases, *P* < 0.0001). Of note, no *CCN* alterations was observed in patients with brain cancers (*P* < 0.001). The biopsy site was more frequently a metastatic site when positive for a *CCN* amplification (18% vs 10%, metastatic vs. primary; *P* = 0.029), Table 1.

### *CCN* amplifications and direct co-alterations

In the overall population, the median number of molecular alterations per patient was four (range 0–16), and it was significantly higher in patients harboring *CCN* alterations (median of eight alterations compared to three in patients without *CCN* alterations, *P* < 0.0001). In a univariate analysis, *CCN* alterations were associated with *FGF/FGFR* alterations (amplification/mutations identified in *FGF3/4/6/10/14/19/23* and *FGFR1/2/3/4*) (*P* < 0.0001), *ZNF217/ZNF703* (*P* < 0.0001), *AURKA* (*P* = 0.001), *ARFRP1*(*P* = 0.003), *EMSY* (*P* = 0.007), and *MDM2* (*P* = 0.030) amplifications, as well as amplification/mutations in *MET* (*P* = 0.016) or *AKT1/2* (*P* = 0.023), Supplemental Table 1.

### Multiple logistic regression analysis of factors associated with *CCN* amplifications

In a multiple logistic regression model (that included any parameters that were significant (*P* < 0.05) in univariate analysis), the only variables that remained statistically associated with *CCN* amplifications were aberrations in *FGF/FGFR* (*P* < 0.0001), *MET* (*P* = 0.003), and *ARFRP1* (*P* = 0.032). The negative association with brain tumors also remained significant (*P* = 0.044) Table 2.

**Table 2:** Multiple logistic regression model for clinical characteristics associated with *CCN* amplifications

Characteristics	*P*-Value	Odds Ratio	95% CI
**Gender (women vs. men)**	0.410	1.58	0.53–4.69
**Histology**			
Breast	0.913	1.06	0.36–3.15
Brain^a^	**0.044**	0.26	0.05–1.51
**Site molecular testing (metastatic vs. primary)**	0.248	1.75	0.68–4.52
**Genomic alterations^b^**			
*FGF/FGFR*	< **0.0001**	41.7	15.9–111.1
*MYC*	0.247	1.93	0.64–5.85
*ZNF217/703*	0.475	1.70	0.40–7.25
*MDM2*	0.267	2.67	0.47–15.15
*AKT1/2*	0.271	2.55	0.48–13.51
*AURKA*	0.390	3.26	0.22–47.62
*ARFRP1*	**0.032**	18.9	1.29–250.0
*MET*	**0.003**	50.0	3.79–689.0
*EMSY*	0.271	6.25	0.24–166.7

a negative association

b *FGF/FGFR* comprised amplification/mutations in *FGF3/4/6/10/14/19/23* and *FGFR1/2/3/4*.

*MYC, ZNF217/703, MDM2, AURKA, ARFRP1, EMSY* alterations were all amplifications. *AKT1/2* and *MET* alterations comprised amplification/mutations.

### *CCN* extended co-amplification network

**Figure 2:**
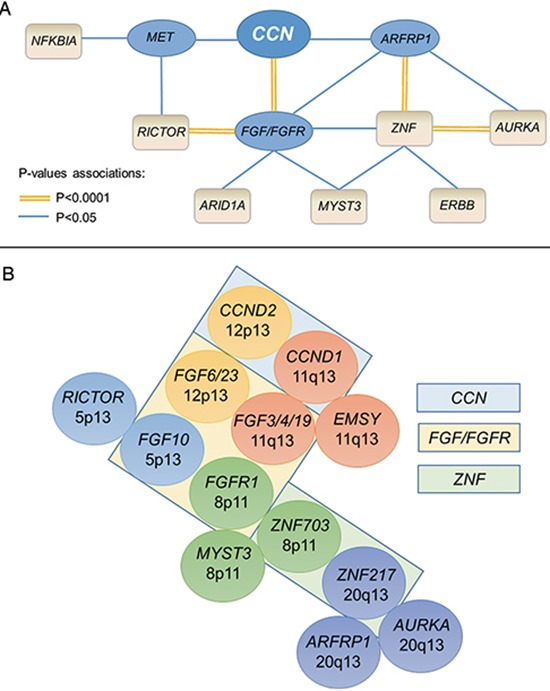
*CCN* and co-alterations In panel A, the connectors represent a statistically significant association in a multiple logistic regression model (*P* < 0.05). The double orange connectors represent a correlation with a *P*-value < 0.0001; in that case, there was chromosomal co-localization (panel B). Cyclin genes were *CCND1, CCND2, CCND3*, and *CCNE1*; *FGF/FGFR* gene family comprised *FGF3/4/6/10/14/19/23* and *FGFR1/2/3/4*; *ERBB* comprised *ERBB2/3/4*; *ZNF* comprised *ZNF217* and *ZNF703*. In panel B, chromosome co-localizations are represented by the same color. Boxes contain genes that are related family members.

We further explored the ramifications of alteration associations, and found, in a multiple logistic regression model, that in addition to its association with *CCN* amplifications (*P* < 0.0001), *FGF/FGFR* associated strongly with *RICTOR* (*P* < 0.0001). *FGF/FGFR* also correlated with *ARID1A* (*P* = 0.033), *ZNF217/703* (*P* = 0.019), *MYST3* (*P* = 0.034), and *ARID1A* (*P* = 0.033). *MET* alterations associated with *RICTOR* (*P* = 0.011) and *NFKBIA* (*P* = 0.032), and *ARFRP1* strongly correlated with both *ZNF217/703* (*P* < 0.0001) and *AURKA* (*P* = 0.006). Lastly, we investigated the associations with *ZNF217/703* amplifications and found strong correlations with *AURKA* (*P* < 0.0001), *ARFRP1* (*P* = 0.002), *FGF/FGFR* (*P* = 0.004), *ERBB2/3/4* (*P* = 0.044), and *MYST3* (*P* = 0.002), Figure 2A and Supplemental Table 2.

Since the alterations with a very strong association (*P* < 0.0001) were amplifications, we examined the chromosome location of these genes and found patterns of co-localization in the same chromosome regions. Indeed, the strong association observed between *CCN* and *FGF/FGFR* family genes can be partly explained by the co-localization of *CCND1* and *FGF3/4/19* at the 11q13 locus. Of note, *EMSY*, that correlated with *CCN* alterations in the univariate analysis, is also found at the 11q13 locus. Additionally, *CCND2* and *FGF6/23* are both localized at 12p13. The strong association observed between *FGF/FGFR* and *RICTOR* can be explained by the co-localization of *FGF10* and *RICTOR* at the 5p13 genomic location. *FGFR1*, *ZNF703* and *MYST3* are localized on 8p11 and *ZNF207* was highly co-amplified with *ARFRP1* and *AURKA* at the 20q13 locus, Figure 2B.

### *CCN* amplifications and clinical outcome

In a univariate analysis, *CCN* amplifications correlated with the development of metastasis in the liver (*P* = 0.005), bone (*P* = 0.036), and brain (*P* = 0.047), Supplemental Table 3. The only association that remained significant in the multiple logistic regression model was the association with the development of liver metastasis (*P* = 0.046). Neither the overall survival, nor the time to metastasis/recurrence were statistically different depending on *CCN* alteration status (HR = 1.17; *P* = 0.646 and HR = 1.08; *P* = 0.691, respectively). No differences in median best progression-free survival time (PFS) or PFS analysis by treatment type was observed when patients with, or without, *CCN* alterations were compared.

### Survival analysis in the overall population

In our population of 392 patients, the median follow up time was 37.8 months (95%CI 30.9–44.8 months). In a univariate analysis, the survival was poorer for patients with gastro-intestinal histology (*P* < 0.0001), men (*P* = 0.036), or patients harboring a *TP53* (*P* = 0.002), *CDK* (*CDK4/6/2/CDKN2A/2B*) (*P* = 0.005), *PTEN* (*P* < 0.0001), or *EGFR* (*P* = 0.030) alterations. Of importance, only the gastro-intestinal histology (*P* < 0.0001), *PTEN* (*P* < 0.0001), and *CDK* alterations (*P* = 0.041) remained statistically significant independent predictors for poorer survival in a Cox regression model, Table 3. *TP53* mutational status was no longer a significant predictor of inferior survival, although a trend persisted with *P* = 0.087. *CCN* was not a survival predictor in univariate analysis (*P* = 0.646).

**Table 3:** Overall survival associations in 392 patients with cancer

Characteristics	Univariable		Multivariable	
	HR (CI 95%)	*P*-Value	HR (CI 95%)	*P*-Value
**Gender (men vs. women)**	0.59 (0.36–0.97)	**0.036**	0.65 (0.38–1.09)	0.107
**Histology**				
Gastro-intestinal (*N* = 91)	0.19 (0.09–0.37)	< **0.0001**	0.28 (0.16–0.49)	< **0.0001**
Breast (*N* = 80)	0.63 (0.37–1.04)	0.073	---	---
Brain (*N* = 56)	1.23 (0.61–2.49)	0.564	---	---
Gynecologic (*N* = 33)	1.47 (0.64–3.38)	0.359	---	---
Head & Neck (*N* = 30)	0.58 (0.25–1.35)	0.207	---	---
Liquid (*N* = 29)	2.37 (0.94–5.93)	0.065	---	---
Melanoma (*N* = 29)	2.23 (0.99–5.02)	0.053	---	---
Lung (*N* = 26)	0.53 (0.17–1.65)	0.269	---	---
Other (*N* = 16)	0.75 (0.16–3.67)	0.722	---	---
**Genetic alteration type^a^**				
TP53 (*N* = 178)	0.47 (0.29–0.75)	**0.002**	0.64 (0.38–1.01)	0.087
CDK (*N* = 104)	0.43 (0.24–0.78)	**0.005**	0.56 (0.32–0.98)	**0.041**
KRAS (*N* = 63)	0.55 (0.27–1.01)	0.089	---	---
FGF/FGFR (*N* = 57)	1.35 (0.74–2.47)	0.324	---	---
PIK3CA (*N* = 53)	1.26 (0.64–2.47)	0.503	---	---
CCN (*N* = 50)	1.17 (0.60–2.26)	0.645	---	---
MYC (*N* = 48)	0.68 (0.33–1.41)	0.295		
PTEN (*N* = 42)	0.09 (0.03–0.23)	< **0.0001**	0.19 (0.10–0.36)	< **0.0001**
EGFR (*N* = 31)	0.29 (0.09–0.89)	**0.030**	0.95 (0.39–2.29)	0.900
BRCA1/2 (*N* = 28)	0.61 (0.24–1.55)	0.300	---	---
APC (*N* = 24)	0.30 (0.09–1.00)	0.051	---	---

a Included alterations with at least 5 events (death) and *N* = 20 patients with the alteration.

*CCN* comprised *CCND1/2/3* and *CCNE1; CDK* comprised *CDK4/6*, *CDKN2A/B*; *FGF/FGFR* comprised *FGF3/4/6/10/14/19/23* and *FGFR1/2/3/4*. Only characteristics that were significant (*P* < 0.05) in the univariable analysis (log-rank test) were included in the multivariable analysis (Cox regression model).

## DISCUSSION

Cyclin amplifications are frequent in cancer. The percentage of cyclin (*CCN*) alteration varies by tumor type; for instance, in previous studies, *CCND1* amplification ranged from 15–40% [1]. Our population had an overall 13% rate of *CCN* alterations, with most (55%) of them being *CCND1* amplifications (Supplemental Figure 1). We found that *CCN* alterations were associated with breast cancer (27% of cases), which is consistent with the literature [1, 21, 22]. CCND1 overexpression was previously found to be associated with shorter patient survival in many cancers and is often correlated with increased risk of metastasis [23, 24]. In our population, the overall survival was not statistically different in the presence of *CCN* abnormalities, perhaps because of the small number of death events at the time of analysis (*N* = 9 deaths amongst 50 patients with cyclin alterations). In a Cox regression model, the variables independently associated with worse survival were gastrointestinal malignancies, consistent with previous publications in advanced cancer [25], and alterations in *PTEN* as well as *CDK* family genes (Table 3). Of note, *TP53* alterations were no longer associated with a shorter overall survival, perhaps because of its association with gastrointestinal histology (*P* = 0.024).

We did not detect any difference in best PFS analysis when patients with, or without *CCN* amplifications were compared. Types of therapy examined comprised regimens that included gemcitabine, taxanes, platinums, bevacizumab, hormonal treatments, and everolimus (though the number of patients treated with the latter drug was small, thus limiting the power of the analysis) (Supplemental Table 3). Of interest, *CCN* alterations were more frequent in metastatic sites (*p* = 0.029; univariate anlaysis), and there was a higher incidence of these alterations in patients who developed liver metastases (multiple logistic regression analysis; *p* = 0.046) (Tables 1 and Supplemental Table 3). Of interest, some previous studies have also described an association between *CCND2* and *CCND3* with liver metastasis [26, 27]. Finally, patients harboring *CCN* amplifications had a median of eight molecular alterations compared to three in patients without *CCN* amplifications (*P* < 0.0001).

Of importance, we uncovered an extended network of co-amplifications in our study. Indeed, *CCN* gene amplifications correlated independently with *FGF/FGFR* (*P* < 0.0001), *ARFRP1* (membrane associated GTPase) (*p* = 0.032) and *MET* alterations (*P* = 0.003) in a multiple logistic regression analysis. The association between *CCN* and *FGFs* is not surprising, considering that *CCND1* and *FGF3*, *4* and *19* co-localize on the same amplicon on chromosome 11q13 [28]. Similarly, *CCND2* and *FGF6* and *23* co-localize on 12p13. A systematic dissection of the genomic associations at different levels (first and second level of association with *CCN* amplifications, Figure 2) revealed a more comprehensive network. Indeed, the co-localization involving *FGFR1* and *ZNF703* has been described [29], consistent with the univariate association between amplifications in *CCN* (which correlates with *FGF/FGFR* aberrations) and *ZNFs*. (*ZNF* products are zinc finger proteins, which bind DNA and, through this binding, regulate gene transcription). *ZNF703*, like *FGFR1*, is localized at the 8p11 locus and there is compelling evidence that *ZNF703* is one of the driver genes in this amplified region [30], especially in breast cancers where *ZNF703* expression seems to be induced by estrogens [31]. *FGFR1* was the first reported oncogene in the 8p11 region, and its amplification and overexpression has been related to poor prognosis and endocrine resistance in breast cancer [32].

Relationships between cyclins and FGF/FGFRs have also been previously reported at the protein level. For instance, a study showed that FGFR4 contributed to the maintenance of CCND1 via the mTOR translation pathway, and several other studies demonstrated cooperation between FGFR and CCND1 [33]. Further, CCND1 can enhance tumor progression, at least in part, by an increased level of transcription of FGFR1 and FGFR2 via E2F [34]. In addition, Kwek et al. [29] suggested that CCND1 induced ZNF703 via the Rb/E2F pathway.

*ZNF217* is localized on 20q13 and can be responsible for enhanced AKT phosphorylation [35]. Of interest, ZNF217 was identified as a marker of poor prognosis in breast cancer [36], was shown to drive epithelial-mesenchymal transition and invasion [36], as well as enhance ERBB3 transcription; the latter is required for ERBB2-dependent breast tumor proliferation, since the ERBB2-ERBB3 heterodimer is the functional “oncogenic unit” [37].

While little is known about ADP-ribosylation factor-related protein 1 gene (*ARFRP1*, anomalies of which correlated with those in *CCN* in multiple logistic regression analysis) and cancer [38], *AURKA*, (Aurora A kinase), which co-localizes with *ARFRP1* and with *ZNF217* on 20q13, appears to be more well characterized and is believed to control the G2/M transition [39]. Anomalies in *AURKA* and *CCN* correlated in our study in a univariate analysis. Finally, *MET* anomalies (which correlated with *CCN* amplifications; multiple logistic regression analysis) correlated with *RICTOR* abnormalities *(*univariate analysis), which in turn co-localized with *FGF10* on 5p13. RICTOR is a component of the second mTOR complex, identified as mTORC2. The mTORC2 can phosphorylate protein kinases including AKT and PKCα, turning on a variety of cellular processes such as proliferation and survival [40].

These co-alterations might, to a certain extent, provide a selective advantage to the tumor. One possibility is that the multiple amplifications in this network cooperate synergistically to increase pathway activation and hence the likelihood of proliferation. Another could be that the contribution of each component to functions other than the cell cycle control may offer an additional selective advantage during oncogenesis. In addition, several studies demonstrated that *CCND1* amplifications were linked to resistance mechanisms, such as endocrine resistance in breast cancers [41, 42], or BRAF inhibitor resistance in *BRAF* V600E mutated melanomas [43]. Further, *CCND1* has also been implicated in acquired radioresistance [44].

Importantly, cyclins have been correlated with positive estrogen receptor (ER+) in breast cancers [28]. In our population, we observed that 31% (17/53) of breast specimens that were ER+ also harbored a *CCN* amplification. Due to the availability of anti-estrogen therapies, ER positivity is considered a marker of good prognosis. However, among patients with ER positive tumors, those also harboring *CCND1* amplification seem to constitute a subgroup with higher grade and a relatively poor prognosis [45, 46]. Recently, the randomized phase 2 trial PALOMA-1 investigating the CDK4/6 inhibitor palbociclib demonstrated a statistically significant and clinically meaningful improvement in progression-free survival (PFS) for the combination of palbociclib and letrozole compared to letrozole alone (20.2 vs. 10.2 months, *P* = 0.0004) in women with ER+/HER2- metastatic breast cancer [47].

Our study had several limitations. First, correlations were made via a retrospective review. As such, the timing of response assessment was performed per the discretion of the attending physician and not completely uniform. Second, diverse histologies were included, and in some there were relatively small numbers of patients. On the other hand, these findings could imply that the impact of cyclin amplifications and co-amplification network is important across histologies. Finally, the molecular testing was done on biopsies obtained at different time points during the clinical course (median of nine months after diagnosis, CI 95% 6–14 months).

*CCN* amplifications appear to have biological implications, including an association with liver metastases, and patterns of molecular correlations, such as those between *CCN* aberrations and *FGF/FGFR* amplifications, as well as *MET* and *ARFRP1* alterations. Further, patients harboring *CCN* amplifications had significantly higher numbers of molecular alterations. The ramifications of a co-amplification network are that these gene products may work together during tumor progression and require tailored combination therapies. As several oncogenic pathways seem to converge on the cyclin-associated pathway and cell cycle progression, the use of drugs to target the cyclin pathway, including CDK4/6 inhibitors, or mTOR inhibitors, have become increasingly relevant, particularly in combinations that can be appropriately customized to attenuate resistance mechanisms.

## METHODS

### Patients

We retrospectively reviewed the clinicopathology and clinical outcomes of 392 patients with advanced cancer (solid and hematologic tumors were considered) who were seen at the UC San Diego Moores Cancer Center and for whom molecular testing had been performed between October 2012 and April 2014. This study was performed and consents obtained in accordance with UCSD Institutional Review Board guidelines.

### Next generation sequencing

Next generation sequencing was performed by Foundation Medicine (FoundationOne™, Cambridge, Massachusetts, http://www.foundationone.com), which is a clinical grade CLIA-approved next-generation sequencing test that sequences the entire coding sequence of 236 cancer-related genes and 47 introns from 19 genes often rearranged in cancer (full list available at http://foundationone.com/genelist1.php). (Nine patients (2.3%) were tested with an earlier version of the panel comprising 182 cancer–related genes). Gene chromosome localization were found using the “genecards” database (http://www.genecards.org/). For the purposes of this analysis, cyclin gene (*CCN*) status was examined for *CCND1, CCND2, CCND3*, and *CCNE1*.

### Statistical analysis

Patients' characteristics were summarized using descriptive statistics. Associations between categorical variables was done using Fisher's exact tests. Time to metastasis/recurrence was defined as the time interval between diagnosis and first metastasis/recurrence, whichever came first. Overall survival (OS) was defined as the time from diagnosis to death or last follow-up date for patients who were alive. Progression-free survival (PFS) was defined as the time from the beginning of a given therapy to progression or treatment discontinuation for any reason. The data for best progression-free survival was available for 246 patients of 392 (63%). Best PFS was defined as the longest PFS achieved on treatment. Patients who did not progress were censored at the last follow up date. Estimations for OS and PFS were done using a Kaplan-Meier analysis, and were compared among subgroups by the log-rank test. Cox regression model were fit to assess the association between OS and patients' characteristics and *CCN* alteration status. Multiple logistic regressions were fit to analyze the association between *CCN* alterations and other patients' characteristics. Only variables with *P*-values less than 0.05 were included in the multiple regression models. All statistical analysis were performed by MS with SPSS version 22.0.

## SUPPLEMENTARY FIGURE AND TABLES


