# Supplementary material for: Cyclin alterations in diverse cancers: outcome and co-amplification network
Source: Oncotarget. 2014 Dec 18;6(5):3033–42. doi: 10.18632/oncotarget.2848 (PMC4413635; doi:10.18632/oncotarget.2848)
Supplement: Supplementary file 1 [file oncotarget-06-3033-s001.pdf]

SUPPLEMENTARY FIGURE AND TABLES

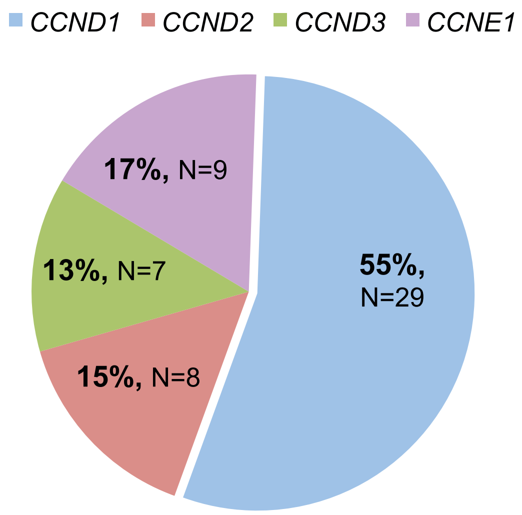

Supplementary Figure 1: Frequency of *CCN* alterations. A total of 53 *CCN* alterations were identified in 50 of 392 patients (13%); all were amplifications; three patients harbored two alterations.

Supplementary Table 1: Coexisting genetic alterations in 392 patients with or without *CCN* amplifications (univariate analysis)

Genetic alterations*	Total patients, *N* = 392	CCN wild- type, *N* = 342	CCN altered, *N* = 50	*P*-value
TP53	179 (45.7%)	150 (43.5%)	29 (58%)	0.069
CDK	104 (26.5%)	87 (25.4%)	17 (34%)	0.230
KRAS	63 (16.1%)	54 (15.8%)	9 (18%)	0.682
FGF/FGFR	57 (14.5%)	22 (6%)	35 (70%)	**< 0.0001**
PIK3CA	53 (13.5%)	44 (12.9%)	9 (18%)	0.374
MYC	49 (12.5%)	38 (11.1%)	11 (22%)	**0.039**
PTEN	42 (10.7%)	36 (10.5%)	6 (12%)	0.806
EGFR	31 (7.9%)	24 (7%)	7 (14%)	0.095
BRCA1/2	28 (7.1%)	22 (6.4%)	6 (12%)	0.149
NF1	27 (6.9%)	24 (7%)	3 (6)	0.540
MCL1	26 (6.6%)	21 (6.1%)	5 (10%)	0.355
APC	24 (6.1%)	22 (6.4%)	2 (4%)	0.753
ARID1A	24 (6.1%)	18 (5.3%)	6 (12%)	0.104
ZNF217/703	23 (5.9%)	12 (3.5%)	11 (22%)	**< 0.0001**
ERBB2/3/4	23 (5.9%)	18 (5.3%)	5 (10%)	0.194
SMAD4	21 (5.4%)	17 (5%)	4 (8%)	0.325
MDM2	20 (5.1%)	14 (4.1%)	6 (12%)	**0.030**
BRAF	19 (4.8 %)	16 (4.7%)	3 (6%)	0.721
RB1	19 (4.8%)	17 (5%)	2 (4%)	1.000
AKT1/2	14 (3.6%)	9 (2.6%)	5 (10%)	**0.023**
NOTCH1	14 (3.6%)	12 (3.5%)	2 (4%)	0.696
ATM	11 (2.8%)	8 (2.3%)	3 (6%)	0.154
AURKA	11 (2.8%)	5 (1.5%)	6 (12%)	**0.001**
NFKBIA	11 (2.8%)	8 (2.3%)	3 (6%)	0.154
MYST3	10 (2.6%)	2 (2%)	3 (6%)	0.123
RICTOR	8 (2%)	6 (1.8%)	2 (4%)	0.271
ARFRP1	6 (1.5%)	2 (0.6%)	4 (8%)	**0.003**
ESR1	6 (1.5%)	4 (1.2%)	2 (4%)	0.171
MET	5 (1.3%)	2 (0.6%)	3 (6%)	**0.016**
EMSY	4 (1%)	1 (0.3%)	3 (6%)	**0.007**

*genes altered in at least 2 patients with *CCN* alterations have been included.
CDK comprised *CDK4/6*, *CDKN2A/B*. *FGF/FGFR* comprised amplification/mutations in *FGF3/4/6/10/14/19/23* and *FGFR1/2/3/4*. *MYC, ZNF217/703, MDM2, AURKA, ARFRP1, EMSY* alterations were all amplifications. *AKT1/2* and *MET* alterations comprised amplification/mutations.

Supplementary Table 2: *CCN* co-amplifications network association (univariate and multivariate analysis)

	Univariate	Multivariate	
	P-Value	*P*-Value	HR (CI 95%)
**Associations with *FGF/FGFR* Parameters:**			
Breast histology	< 0.0001	0.110	0.47 (0.18–1.19)
CCN	< 0.0001	**< 0.0001**	0.03 (0.01–0.08)
ZNF217/703	< 0.0001	**0.019**	0.11 (0.02–0.70)
EMSY	0.012	0.767	0.65 (0.04–11.4)
MDM2	0.012	0.075	0.27 (0.07–1.14)
AKT1/2	0.031	0.225	0.36 (0.07–1.87)
ARFRP1	0.029	0.417	2.92 (0.22–38.8)
ARID1A	0.002	**0.033**	0.24 (0.07–0.89)
ATM	0.008	0.136	0.28 (0.05–1.50)
AURKA	0.001	0.482	2.19 (0.25–19.4)
ERBB2/3/4	0.007	0.509	0.63 (0.16–2.45)
MYST3	0.001	**0.034**	0.12 (0.02–0.85)
NFKBIA	0.049	0.310	0.39 (0.06–2.40)
RICTOR	0.002	**< 0.0001**	0.03 (0.01–0.16)
**Associations with *ARFRP1* Parameters:**			
Breast histology	0.006	0.166	0.12 (0.01–2.71)
CCN	0.002	**0.035**	0.08 (0.003–2.02)
FGF/FGFR	0.029	**0.048**	0.67 (0.00–0.92)
ZNF217/703	< 0.0001	**< 0.0001**	0.01 (0.00–0.32)
EMSY	0.009	0.174	0.21 (0.01–6.22)
MYC	0.016	0.230	0.36 (0.03–5.27)
AURKA	< 0.0001	**0.006**	0.09 (0.003–0.17)
BRCA	0.030	0.103	0.002 (0.00–0.75)
SMAD4	0.012	0.068	0.03 (0.001–0.79)
**Associations with *MET* Parameters:**			
Lung	0.014	0.159	0.15 (0.01–2.13)
CCN	0.010	0.062	0.10 (0.01–1.12)
CDK	0.030	0.065	0.07 (0.004–1.18)
MYC	0.009	0.086	0.12 (0.01–1.35)
EFGR	0.024	0.739	0.64 (0.05–8.83)
NFKBIA	0.048	**0.032**	0.03 (0.001–0.74)
RICTOR	0.0002	**0.011**	0.03 (0.002–0.43)

(*Continued*)

	Univariate	Multivariate	
**Associations with *ZNF217/703* Parameters:**			
Breast histology	< 0.0001	0.059	0.19 (0.04–1.06)
CCN	< 0.0001	0.630	1.54 (0.27–8.88)
FGF/FGFR	< 0.0001	**0.004**	0.04 (0.01–0.37)
EMSY	0.001	0.116	0.02 (0.00–2.63)
MYC	0.011	0.303	0.39 (0.07–2.34)
PIK3CA	0.019	0.876	1.16 (0.18–7.40)
ARFRP1	< 0.0001	**0.002**	0.01 (0.00–1.14)
ARID1A	0.029	0.805	0.73 (0.06–9.21)
AURKA	< 0.0001	**< 0.0001**	0.00 (0.00–0.01)
ERBB2/3/4	0.003	**0.044**	0.13 (0.02–0.94)
MYST3	< 0.0001	**0.002**	0.04 (0.004–0.30)

Genomic alterations with *P*-values less than 0.05 are shown in the table for the univariate analysis (left side) and were consequently all included in the multivariate analysis (right side, multiple logistic regression model). *CCN* genes comprised *CCND1, CCND2, CCND3*, and *CCNE1*. *FGF/FGFR* gene family comprised *FGF3/4/6/10/14/19/23*, and *FGFR1/2/3/4*. *CDK* genes comprised CDKN2A/B and CDK4/6.

Supplementary Table 3: Clinical outcomes of 392 patients with or without *CCN* amplifications

Outcomes	Total patients, $N = 392$	*CCN* wild- type, $N = 342$	*CCN* altered, $N = 50$	*P*-value (univariate)	*P*-value (multivariate)
**Metastasis site[1]**					
Lymph node	141	124 (88%)	17 (12%)	0.875	–
Liver	98	77 (79%)	21 (21%)	**0.005**	**0.046**
Lung	79	69 (87%)	10 (13%)	1.000	–
Bone	79	63 (80%)	16 (20%)	**0.036**	0.236
Brain	32	24 (75%)	8 (25%)	**0.047**	0.129
Peritoneal	49	45 (92%)	4 (8%)	0.368	–
Soft tissue	31	28 (90%)	3 (10%)	0.782	–
Other[2]	40	37 (93%)	3 (7%)	0.451	–
**Best PFS*[a] (Median, months)**					
Gemcitabine	6.7, $N = 35$	6, $N = 31$	7.6, $N = 4$	0.966	
Everolimus	3.4, $N = 7$	3.4, $N = 4$	4.8, $N = 3$	0.607	
Hormonal therapy[3]	10.3, $N = 22$	10.3, $N = 14$	11.0, $N = 8$	0.948	
Taxane-based regimen[4]	8.4, $N = 38$	8.4, $N = 27$	7.6, $N = 11$	0.944	
Capecitabine/5-FU	7.2, $N = 55$	7.2, $N = 47$	7, $N = 8$	0.970	
Platinum-based regimen[5]	7, $N = 67$	7, $N = 55$	8, $N = 12$	0.588	
Bevacizumab	8.7, $N = 41$	8.7, $N = 38$	4, $N = 3$	0.837	
**All PFS*[a] (Median, months)**					
Everolimus	3.5, $N = 30$	4, $N = 21$	3.5, $N = 9$	0.806	
Hormonal therapy[3]	9.5, $N = 35$	9.5, $N = 22$	8.7, $N = 13$	0.501	
Taxane-based regimen[4]	6.7, $N = 71$	5.5, $N = 57$	7.6, $N = 14$	0.572	
Capecitabine/5-FU	6, $N = 81$	6.9, $N = 67$	5.8, $N = 14$	0.568	
Platinum-based regimen[5]	5, $N = 100$	5, $N = 71$	5.8, $N = 19$	0.884	

[1]Patients may have multiple sites of metastasis.
[2]Adrenal, $n = 12$; Colon, $n = 8$; kidney, $n = 4$; ovary, $n = 3$; pancreas, $n = 7$; skin, $n = 6$
[3]Hormonal therapy were defined as exemestane, tamoxifen, fulvestrant, or anastrazole containing regimen.
[4]Taxane regimens comprised paclitaxel, docetaxel, or abraxane.
[5]Platinum-based regimen comprised cisplatin, oxaliplatin, or carboplatin.
*Adjuvant/neoadjuvant therapies have been excluded. Some patients have been treated with several therapies described and there is overlap.
[a]any regimen that contained the mentioned drug was included.

Supplementary Table 4: Outcome comparison according to *CCN* amplifications

Endpoint	HR	CI 95%	*P*-value
Time to metastasis/recurrence	1.08	0.75–1.54	0.691
Overall survival	1.17	0.60–2.26	0.646
Median Best PFS	1.11	0.47–1.75	0.291
